# Japanese Dentistry

**Published:** 1869-06

**Authors:** 


Japanese Dentistry.—They have dentists in Japan, who evidently do not enjoy the benefits of Dental Associations and journals. The Japanese are a remarkable people; their jugglers are unsurpassed; but commend us not to their dentists. Their manner of extracting a tooth must be tempting to their patients, and reminds one of the method of removing a rusty screw. The tooth is tapped with a mallet, until it can be extracted with the fingers; pleasantly suggestive of an amount of malleting which we should think would not commend Japanese dentistry.

